# Reliability and validity of DTI-based indirect disconnection measures

**DOI:** 10.1016/j.nicl.2023.103470

**Published:** 2023-07-11

**Authors:** A.R. Smits, M.J.E. van Zandvoort, N.F. Ramsey, E.H.F. de Haan, M. Raemaekers

**Affiliations:** a UMC Utrecht Brain Center, Department of Neurology and Neurosurgery, University Medical Center Utrecht, the Netherlands; b Department of Psychology, University of Amsterdam, the Netherlands; c Department of Experimental Psychology, Helmholtz Institute, Utrecht University, the Netherlands; d St. Hugh's College, Oxford University, United Kingdom

**Keywords:** Diffusion tensor imaging (DTI), Disconnection, Stroke, Prediction, Validity, Reliability

## Abstract

White matter connections enable the interaction within and between brain networks. Brain lesions can cause structural disconnections that disrupt networks and thereby cognitive functions supported by them. In recent years, novel methods have been developed to quantify the extent of structural disconnection after focal lesions, using tractography data from healthy controls. These methods, however, are indirect and their reliability and validity have yet to be fully established. In this study, we present our implementation of this approach, in a tool supplemented by uncertainty metrics for the predictions overall and at voxel-level. These metrics give an indication of the reliability and are used to compare predictions with direct measures from patients’ diffusion tensor imaging (DTI) data in a sample of 95 first-ever stroke patients. Results show that, except for small lesions, the tool can predict fiber loss with high reliability and compares well to direct patient DTI estimates. Clinical utility of the method was demonstrated using lesion data from a subset of patients suffering from hemianopia. Both tract-based measures outperformed lesion localization in mapping visual field defects and showed a network consistent with the known anatomy of the visual system. This study offers an important contribution to the validation of structural disconnection mapping. We show that indirect measures of structural disconnection can be a reliable and valid substitute for direct estimations of fiber loss after focal lesions. Moreover, based on these results, we argue that indirect structural disconnection measures may even be preferable to lower-quality single subject diffusion MRI when based on high-quality healthy control datasets.

## Highlights

• Brain lesions can cause structural disconnections and network disruption.
• Normative tractography data is used to quantify structural disconnection, implemented in a tool.
• Reliability of this approach is evaluated in first-time stroke patients with or without visual field defects.
• Tool-based predictions provide a valid substitute for direct estimations of fiber loss from from patient’s DTI.

## 1 Introduction

Studies of patients with focal brain lesions, particularly stroke, have yielded valuable insights into brain-behaviour relationships. Technological advancements in neuroimaging and statistics now allow researchers to investigate lesion data on a large scale and renewed interest for lesion-symptom mapping in recent years (Bates et al., 2003, Karnath et al., 2018). Generally, these studies are conducted for either of two goals: to identify structural correlates underlying a function of interest, or to predict stroke outcome or recovery (Price et al., 2017). Previous studies identified cortical areas critically involved in, for example, spoken language (Mirman et al., 2015), visuospatial processing (Baier et al., 2012, Biesbroek et al., 2014) and memory (Lugtmeijer et al., 2021, Mock et al., 2022). In addition, prediction studies showed relative contributions of lesion location and volume to, for example, post-stroke aphasia (Forkel and Catani, 2018, Fridriksson et al., 2015, Schumacher et al., 2019) and neglect recovery (Karnath et al., 2011).

While traditional lesion symptom mapping studies contributed greatly to the field of neurology and neuroscience, lesion location alone is not the optimal proxy for a functional deficit (Catani and Ffytche, 2005, Fornito et al., 2015). This is especially true when the affected area includes white matter, as damage to any location along the length of an axon will induce a similar functional deficit. In theory, fiber tractography based on diffusion weighted imaging could address this issue, as this can be used to estimate white matter tracts in the brain and reconstruct the brain’s structural connectome. This method has also been applied to stroke patients (Bonilha et al., 2014, Forkel et al., 2014, Lunven et al., 2015, Rosenzopf et al., 2022), but the quantification of white matter damage is challenging (Jeurissen et al., 2019), especially in lesioned brains. Also, diffusion weighted imaging of sufficient quality for fiber tractography is often not available from clinical data. An alternative method to quantify network damage is to use tractography data from healthy controls to simulate network effects of lesions.

In the last five years, several research groups have followed this line of reasoning and developed methods for the quantification of disconnection after stroke (Bowren et al., 2022, Foulon et al., 2018, Griffis et al., 2021, Kuceyeski et al., 2015, Salvalaggio et al., 2020). The workflow of these methods is as follows. First, lesions of individual (stroke) patients are delineated on MRI or CT images providing binary lesion maps. These maps are then transformed to a common coordinate system and used in a tractography atlas or database based on healthy controls, to assess which fibers cross the lesioned area. Together, these studies showed that it is possible to quantify white matter disconnection using tractography data from healthy controls and provided early positive results about the clinical utility of these measures. Fiber tracking is a complex endeavor and the resulting tractogram will differ depending upon the quality of the acquired diffusion MR dataset. Obtaining high quality diffusion MR in patients is difficult due to the long acquisition time that is needed, and not all medical centers have the high-end MRI scanners required to do this. Using predictions of structural disconnection based on diffusion datasets from healthy subjects allows for the use of the highest possible angular and spatial resolution. However, the amount of noise, i.e., to what extent these predictions deviate from direct results based on acquisitions in patients, that is introduced in this step is still largely unknown.

In this study, we present a new tool to predict the impact of a lesion on the structural connectome using a technique based on diffusion datasets from healthy subjects. These predictions are supplemented with metrics of uncertainty, both for the overall predictions, and at the level of individual voxels. These metrics allow us to assess whether indirect measures of structural disconnection, as provided by the tool, are comparable to direct measures from patient’s DTI. In addition, the metrics provide an indication of the reliability of the tool. This is achieved by quantifying differences between tool predictions and the results based on DTI data of patients, relative to the estimated prediction uncertainties. Taken together, these analyses will convey whether indirect methods can be a reliable substitute for DTI from patients for structural disconnection mapping. In addition, we performed the analyses with healthy control diffusion data from different databases in order to gain better insight in parameters driving the quality of the predictions, and established the effect of lesion size on the reliability of predictions. Finally, we perform validation by predicting white matter loss in a subset of patients who suffered from hemianopsia, and assess whether the results match with the known anatomy of the visual system as a ground-truth model.

## 2 Methods

### 2.1 Overview

The predictions of damage to the white matter structure by a lesion are created in MNI space and are made by superimposing a lesion volume of a patient on a tractogram of a healthy control subject, where tracks (or streamlines) passing through the lesion represent the predicted damage to structural connectivity. By repeating this step for multiple tractograms of control subjects in a database, mean predictions and their uncertainty can be assessed in 3D MNI space.

### 2.2 Patient data

The patient data reported in this paper were collected as part of the Functional Architecture of the Brain for Vision (FAB4V) project (Lammers et al., 2022, Lugtmeijer et al., 2021). The project was approved by the UMC Utrecht institutional ethical board in accordance with the declaration of Helsinki (Association, 2013) and written informed consent was obtained prior to participation. Inclusion criteria were (1) clinical diagnosis of ischemic stroke, (2) age between 18 and 90, and (3) fluent in Dutch. Presence of a comorbid neurological, psychiatric, or other condition that may interfere with cognitive testing/imaging was reason for exclusion. A subset of 95 adult patients with a first-ever symptomatic ischemic stroke, a confirmed ischemic lesion on MRI, and with availability of diffusion MRI data was included in the current study. Data of this subset were gathered in Amsterdam University Medical Center (AMC) and University Medical Center Utrecht (UMC Utrecht) in the Netherlands between September 2015 and January 2020. Imaging was performed between 3 weeks and 3 months post-infarction with an average interval of 8 weeks. Demographics of this cohort are described in Table 1 and a lesion overlay is displayed in supplementary Fig. 1.

**Table 1:** Acquisition parameters of the different diffusion datasets used in the experiments.

	HCP	UMCU-1	UMCU-2	Patients
Age (SD)	29 (3)	46 (21)	46 (21)	55 (15)
Sex (%male)	39.3	48.1	48.1	67.4
Diffusion volumes	286	64	32	34
Resolution (mm)	1.10 × 1.10 × 1.05	1.87 × 1.87 × 2.00	1.87 × 1.87 × 2.00	2.00 × 2.00 × 2.00
Nr slices	132	75	75	60
Datasets	173	78	78	95
Field strength	7 T	3 T	3 T	3 T
Headcoil Channels	32	32	32	32
TR (ms)	7000	7110	7110	7498
TE (ms)	71	69	69	90
Multiband factor	2	NA	NA	NA
B-values (s/mm2)	1000/2000	1000	1000	1000
Series	2	2	2	1
Lesion location				
%left				54
%right				41
%bilateral				5
Lesion volume (ml)				17.8 (3.7)

### 2.3 Data inclusion

We used several diffusion MRI datasets in this study. These included a dataset of 78 healthy control subjects acquired at the University Medical Center Utrecht (UMCU), the 7 T diffusion data of 173 healthy control subjects that were scanned as part of the Human Connectome Project (Vu et al., 2015), and 95 datasets of stroke patients from the FAB4V project. Datasets included structural images in addition to the diffusion data, which consisted of an MPRAGE for healthy controls, and a FLAIR for stroke patients. Note that whereas the HCP and UMCU datasets had two complete diffusion weighted series with opposing phase encoding blip, the patient data includes only one complete diffusion series. The second series of the patient data only included a b = 0 image. This means that the number of diffusion images that is acquired is almost halved in the patient dataset, which would lead to less reliable diffusion estimates. To mimic the patient data as closely as possible for optimal comparison, we created an additional database based on the UMCU data, where of the second diffusion series only the b = 0 image was used. This resulted in 3 databases with tractograms of healthy control subjects, which were the HCP (based on the Human Connectome Project data), UMCU 1 (2 phase encoding blips), and UMCU 2 (1 phase encoding blip). A summary of the acquisition parameters of the different datasets can be seen in Table 1.

### 2.4 Processing of DWI data of patients/control subjects

For analysis of the DWI-data we used a combination of FSL (FMRIB, Oxford, UK), MRtrix (https://doi.org/10.1016/j.neuroimage.2019.116137), SPM12 (https://fil.ion.ucl.ac.uk/spm/), and custom scripts in the Interactive Data Language (IDL, Harris Geospatial Solutions, Colorado, USA). DWI data was denoised with ‘dwidenoise’ in MRtrix (Veraart et al., 2016a, Veraart et al., 2016b), and geometry corrected using the B = 0 images of the series with opposed traversal of k-space in the phase-encode direction using FSL’s ‘topup’ (Andersson et al., 2003). DWI data were then corrected for eddy currents and head motion (Andersson and Sotiropoulos, 2016) using the multi-processor variant of FSL's ‘eddy’. A mean B0-image was calculated and stored as reference for registering other images to the DWIs. A response function for constrained Spherical Deconvolution (CSD) was created with ‘dwi2response’ of MRtrix, based on the diffusion data (Dhollander et al., 2018, Dhollander et al., 2016), and subsequently applied for estimating the fiber orientation distribution (FOD) for each voxel with MRTrix’s ‘dwi2fod’ (Tournier et al., 2004).

5 million streamlines were generated for each subject using the deterministic Constrained Spherical Deconvolution algorithm of the ‘tckgen’ function in MRTRix (Tournier et al., 2012), with a stepsize of 0.1 times the voxelsize, a minimum streamline length of 5 times the voxelsize, a tracking cutoff at an FOD of 0.1, and a maximum angle of 9 degrees per step. Seeds were picked randomly from the white matter. In order to improve biological plausibility of the tractograms, the 5 million streamlines were filtered down to 1 million using ‘tcksift’ of MRTrix, by removing streamlines so that the FOD lobe integrals better match the streamline densities (Smith et al., 2013).

For normalizing the tractograms to MNI space, they were first registered to the structural images using parameters based on registration using the B = 0 images. Displacement maps for moving the coordinates of the tractograms of healthy control subjects to MNI space were obtained based on the T1-weighted images and unified segmentation (‘SEGMENT’) in SPM12 (Ashburner and Friston, 2005). For obtaining these maps for patients, we used the clinical toolbox, which also makes use of unified segmentation and multi-tissue templates, and allows normalization to MNI while ignoring voxels that enclose structural abnormalities (Rorden et al., 2012). As input for the clinical toolbox we selected the mask of the manually delineated lesion and the FLAIR image. To avoid systematic misalignments between subjects in MNI-space, we used the same multi-tissue template for both patients and control subjects. We chose the elderly template that is part of the clinical toolbox, which would conceptually cope better with elderly patients with substantially enlarged ventricles. To further improve alignment, and to reduce bias as the result of the use of a single template, the warping regularization was reduced by an order of magnitude (relative to default). The resulting displacement maps were then applied to the coordinates of the tractograms to warp the streamlines into MNI space using a custom script in IDL.

### 2.5 Predictions of damage to the white matter structure

**Fig. 1:**
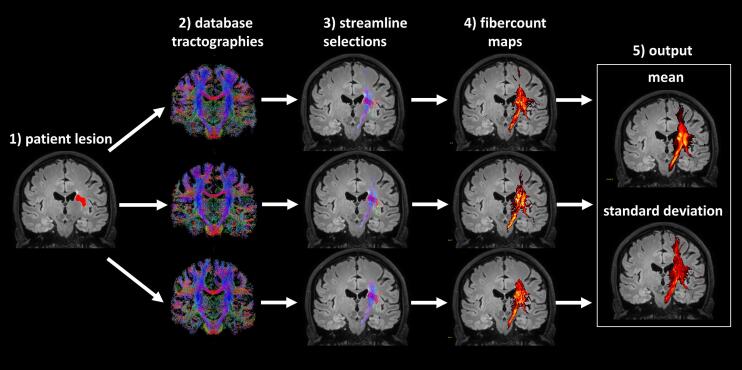
Schematic of the procedure for creating predictions of damage to the white matter structure. A lesion was manually segmented and normalized to MNI space (1), and subsequently imposed on normalized tractographies of control subjects in a database (2). For each subject in the database all streamlines that touched the lesion area were selected (3), and mapped to a volume representing the fiber count for each voxel (4). The mean and standard deviation of the fiber count maps was calculated across subjects in the database (5).

The white matter fibers predicted to be damaged by the stroke were selected by including streamlines from the tractograms for which at least one coordinate was positioned within a voxel marked as part of the lesion. The result of this streamline selection was subsequently used to create a volumetric visitation map representing the number of damaged streamlines passing each voxel. These volumes were created for every subject in the database, after which the mean and standard deviation across the subjects was calculated. The mean represents the prediction, and the standard deviation the uncertainty of the prediction, whose inverse could theoretically be used as a weighting factor in a second level analysis. A schematic representation of the method for creating predictions can be seen in Fig. 1. The reliability of the results was further assessed by performing a leave-one-out cross validation, where each individual control subject result was Pearson correlated with the mean of all other control subjects in the database. An MNI whole brain mask was used to select voxels to be included for calculating the correlation coefficients. The results were averaged after Fischer transformation, and the result was inverse Fischer transformed. The cross-validation result is further referred to as the *internal* reliability of the prediction. The voxels within the lesion area are included for each subject per definition and the leave-one-out-correlation within this area is likely to be higher than the overall internal reliability. This may lead to an overestimation of the reliability in larger lesions. To account for this, the internal reliability was calculated both with inclusion and exclusion of the lesion area.

### 2.6 Comparison with patient DWI results

The predictions of damage to the white matter structure were compared to those as estimated through the tractograms that were created based on the DWI data of the patients. Note that the most accurate benchmark for comparison are the predictions based on the UMCU 2 database, as it included DWI data most closely matching those of the patients. Streamlines in patients were assessed as damaged if they either had a single coordinate within a voxel that was part of the lesion or had a starting point in the area directly surrounding the lesion. The surrounding area was defined through a 2 mm dilation of the lesion area, and was included to avoid missing streamlines that terminate before the edge of the lesion due to partial voluming effects. The selected streamlines were subsequently used to create a visitation map that was correlated to the predictions based on the databases. Similarly for measuring the internal reliability, voxels for calculating the correlation coefficients were selected based on a whole-brain MNI mask. Additional masking was applied if the field of view of the DWI acquisition in the patients did not include the entire brain. This correlation value is termed the *external* reliability of the prediction. Importantly, neither for estimating the internal reliability, nor for the external reliability smoothing was performed on the fiber-count maps, as the reliability scores are intended to reflect the accuracy of the predictions relative to an individual tractography.

To assess the validity of the standard deviations of the predictions, we assessed for each patient if the values of their visitation maps were on average within the range that would be anticipated based on the prediction mean, standard deviation and standard error. For this we first calculated the difference maps, including for each voxel the difference between the prediction and the fiber-count maps of the patients. Then we calculated the ratio between the observed and predicted deviation at each voxel, thereby producing normalized differences from the prediction. Finally, the standard deviation from zero across the normalized differences was calculated. We term this value the *deviation-score*, which was benchmarked against its theoretical value of 1, in case of fully accurate standard deviations. In group-wise analyses we assessed deviation scores and differences between internal and external reliabilities, and determined to what extend these were determined by the used database and lesion size. Differences in reliabilities and deviation scores were tested using repeated measures General Linear Models (GLMs) and post-hoc paired samples t-tests. When necessary, the degrees of freedom of the GLM were adjusted for non-sphericity using the Greenhouse-Geiser method. All group-wise statistical analyses of reliabilities were done after Fischer-transformation of individual correlation coefficients to z-scores. All group-wise reliabilities reported in the figures and text are the result after inverse Fischer transformation of group-mean z-scores back to correlation coefficients. A positive evaluation of the method would include absence of differences between internal and external reliabilities, and deviation scores not exceeding 1 for the UMCU 2 database. This would provide an indication that predictions based on healthy controls would perform equally well as predictions based on diffusion data of patients, when used in a second-level analyses.

### 2.7 Example of application in a lesion symptom mapping approach

To assess the applicability of the tool's predictions in a lesion symptom mapping approach and compare them with predictions based on patients DWI data, we divided the patients in 2 groups, those diagnosed with a stroke-induced visual field defect (n = 11) and those without (n = 84). Five pseudo independent-samples *t*-test were implemented in SnPM (https://nisox.org/Software/SnPM13/) comparing two groups, with subsequently using the white matter damage predictions of the 3 databases, the lesion maps, and the visitation-map based on the patient’s DTI as input. The visitation maps based on the patient’s DTI were smoothed with a kernel of 4 mm FWHM, to account for error in the normalization to MNI. Note that no smoothing was performed on the white matter damage predictions, as these are inherently smooth due to being an average of many subjects. To allow merging of the patients with visual field defects in a single group, the images of patients with a right visual field defect (n = 4) were flipped over the left/right (x)-axis, which was also done randomly for a similar percentage (n = 31) of patients without a visual field defect. A maximum of 5000 permutations were computed for statistical inference, with the threshold set at α = 0.05 (One-sided, Family Wise Error). One sided testing was performed as no voxels with an inverse relationship between streamline damage and visual field defects were anticipated. This rationale is similar to that of the typical lesion-symptom mapping approach (Shahid et al., 2017). If such voxels exist, this is most likely due to patients without visual field defects being more likely of having damaged streamlines outside the visual system.

## 3 Results

### 3.1 Reliability of the predictions

**Fig. 2:**
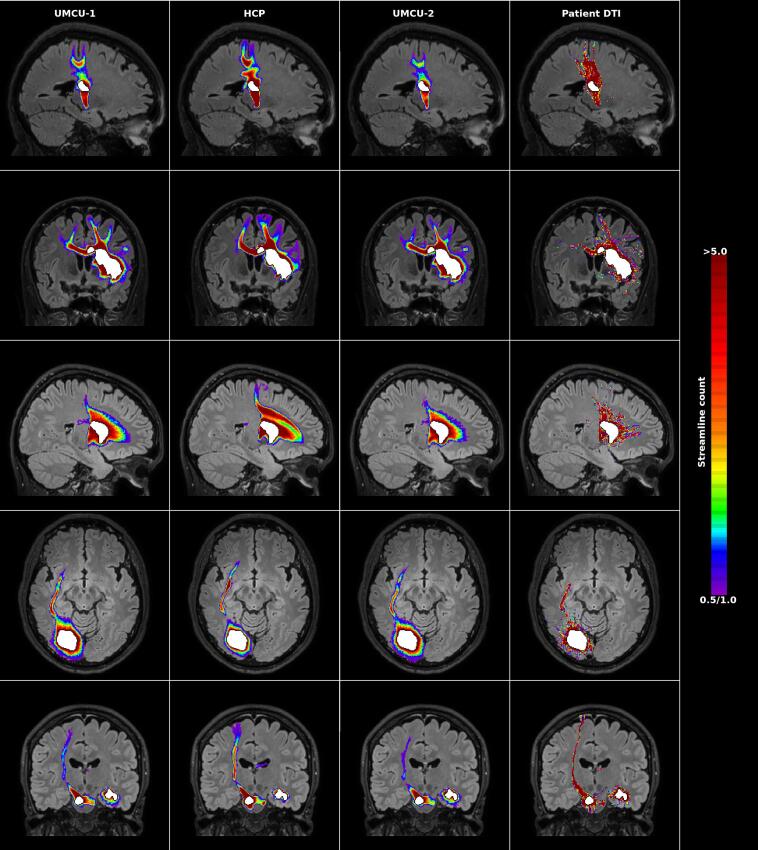
Examples of predictions of white matter damage following stroke. The left three columns depict the predictions by the three databases, where the density of damaged streamlines (count ≥ 0.5) is superimposed on the normalized FLAIR image of the individual patient. The right column shows the fiber-counts of streamlines touching the lesion, or starting in the perilesional area according to the diffusion data of the patients (count ≥ 1). Each row provides the results for a different patient. The segmented lesion is shown in white. For each patient the most informative slice is shown (i.e. the slice including on average the maximum extent of the prediction). Images are displayed according to the neurological convention.

**Fig. 3:**
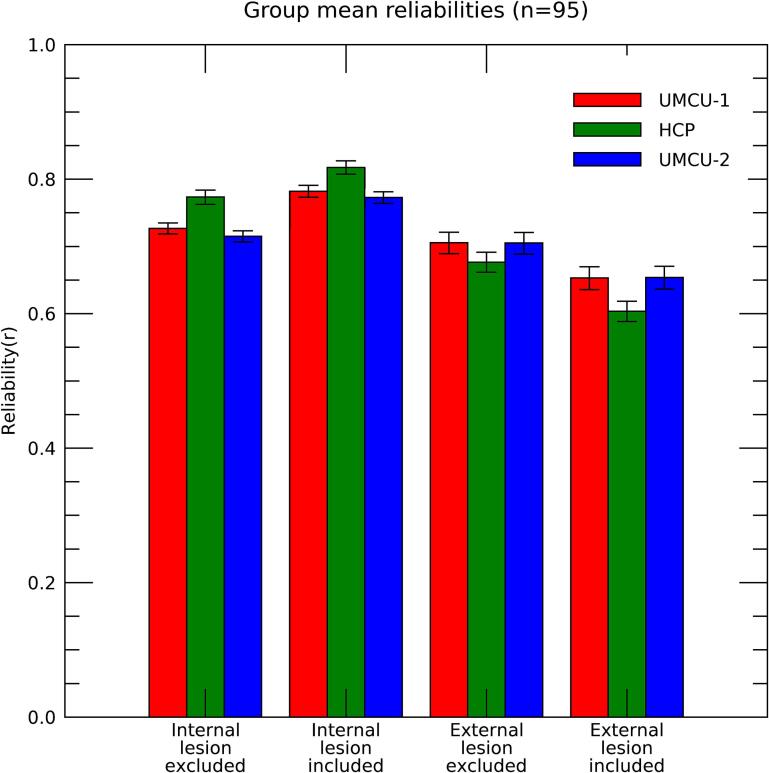
Group mean internal and external reliabilities for the three databases, calculated with inclusion and exclusion of the lesion area. Bars indicate the 95% confidence intervals. The benchmark for the validity of reliabilities of the predictions is provided by the comparison between the internal and external reliability of UMCU 2 with the exclusion of the lesion area.

Fig. 2 shows several examples of the predictions of white matter damage, including the results obtained using the diffusion data of the patients as reference. The group-mean reliabilities of the predictions are depicted in Fig. 3, and were analyzed using a repeated measures GLM with 3 within-subject factors including database (3 layers: UMCU 1, HCP, UMCU 2), lesion (2 layers: included, excluded), reliability (2 layers: internal, external). All main and interaction effects were statistically significant (p < 0.01).

#### 3.1.1 Indirect versus direct measures of tract damage

The primary benchmarking of the predictions is represented by the comparison between the internal and external reliabilities of UMCU 2 (the database using diffusion data most similar to that of the patients). However, this comparison can potentially be influenced by disruptions of tractographies in the lesion area of patients, as these would introduce additional differences between tool predictions and patient results. Indeed, including the lesion area enhanced the internal reliability, but attenuated the external reliability (F_(1,94)_ = 612.67; p < 0.001), indicating that an unbiased comparison between the internal and external reliabilities of UMCU 2 can only be done with exclusion of the lesion area. Results of this benchmark indicated no difference between the internal and actual reliability (Mean internal reliability = 0.715; Mean external reliability = 0.705; paired-t_(94)_ = 1.518; p = 0.132). Note that while this finding does not proof that no differences exist, they give an indication that predictions of white matter damage based on diffusion data of patients do not substantially outperform predictions based on diffusion data of healthy control subjects.

Interestingly, although the number of diffusion volumes included in individual datasets used for the UMCU 1 database was double that of those included in UMCU 2, the external reliabilities for these two databases (excluding lesion area) were nearly identical (Mean UMCU 1 = 0.706; mean UMCU 2 = 0.705; paired-t_(94)_ = 0.726; p = 0.470). Furthermore, the spatial correlations between the predictions of UMCU 1 and UMCU 2, proved that they had a highly similar pattern (group-mean correlation = 0.986; negligible confidence intervals), further indicating that the higher quality of the used diffusion datasets did not substantially impact prediction results. However, the internal reliabilities were higher for UMCU 1 than for UMCU 2 (mean UMCU 1 = 0.727; Mean UMCU 2 = 0.715; paired-t_(94)_ = 11.85; p < 0.001). This finding most likely reflects limitations in the use of individual DTI datasets as a gold standard, a topic which is further addressed in the discussion.

#### 3.1.2 The effect of diffusion MRI data quality

Differences in performance across databases consisted primarily of an interaction between type of reliability (internal/external) and database (F_(2,98)_ = 219,36; p < 0.001), which was largely caused by a higher internal reliability for the HCP than the UMCU databases, while the reverse was true for the external reliability (F_(1,94)_ = 221.048; p < 0.001). These effects are in all likelihood caused by the large differences in acquisition parameters of the diffusion data underlying HCP compared to the other databases. Most likely the enhanced quality of diffusion data and its subsequent impact on the tracking algorithm would minimize between-subject variations of tractographies, and thus enhance the internal reliability of the HCP database. The qualitative nature of the HCP results would, however, be different from those based on diffusion data of the patients, e.g., due to a better performance in resolving crossing fibers, resulting in a lower external reliability. We thus would expect the reductions in external reliability for HCP to be absent if the same acquisition parameters would have been used in patients, and use of the HCP database might still prove to be most advantageous.

#### 3.1.3 The effect of lesion size

**Fig. 4:**
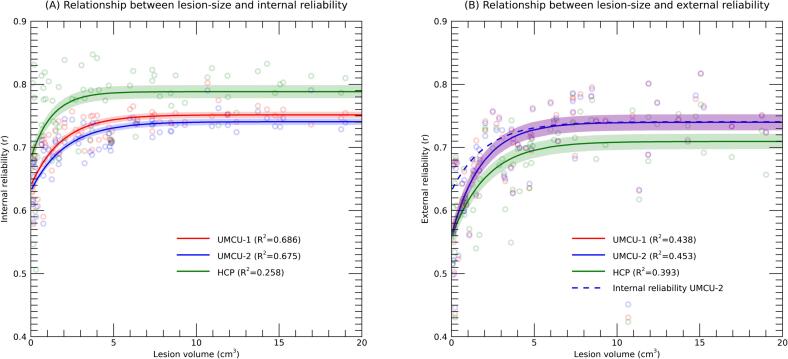
Internal (A) and external (B) reliabilities plotted against the volume of the lesion, with the different colors indicating the different databases used. Each dot is the result of a single patient and database. Solid lines represent the results of an exponential fit, with adjacent shaded areas marking the 95% confidence intervals. The R^2^ in the legend represents the proportion of variance explained by the polynomial fit, adjusted for the number of parameters in the model. The x-axis is truncated at 20 cm^3^ for clearer visualization, excluding 20 patients from the plot (but not the analysis). Fig. 3B includes the exponential fit of the internal reliability of UMCU 2 as reference.

We explored the influence of lesion volume on internal and external reliabilities. This relationship was only assessed with exclusion of the lesion area, to avoid direct artefactual inflation of reliabilities through variations in overlap of the lesion volume. An exponential curve was fitted to the data, which for all databases showed decreased reliability for smaller lesions, both for internal and external reliabilities (Fig. 4). Importantly, the curves for the internal and external reliabilities of UMCU 2 were overlapping, except for the smaller lesions (approximately ≤ 5 cm^3^). Overestimation of the reliability of predictions was thus driven by patients with smaller lesions.

### 3.2 Validity of standard deviations

**Fig. 5:**
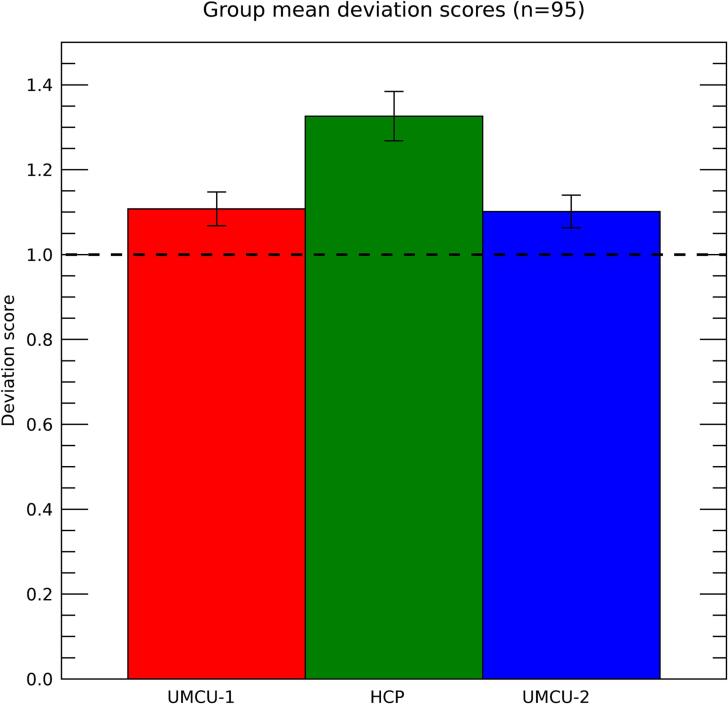
Deviation-scores for the different databases. Bars show the 95% confidence intervals. The horizontal dashed line at a deviation-score of 1.0 is the reference for a fully realistic estimate of standard deviations provided by the prediction.

A deviation-score was assessed for each patient, where a score of 1 reflects an accurate prediction of standard deviations, and a score exceeding 1 reflects that standard deviations are underestimated. The deviation-scores when using the different databases are depicted in Fig. 5. They were larger than 1 for all 3 databases, including UMCU 1 (mean = 1.107; one-sample t_(94)_ = 5.373; p < 0.001) HCP (mean = 1.32; one-sample t_(94)_ = 11.126; p < 0.001), and UMCU 2 (mean = 1.101; one-sample t_(94)_ = 5.235; p < 0.001). Furthermore, a repeated measures GLM with database as within-subject factor (3 layers; UMCU 1, HCP, UMCU 2) showed that the deviation-scores were substantially higher for HCP relative to the two other databases (F_(1,94)_ = 64.312; p < 0.001). These relatively attenuated values for HCP are expected due to the differences in acquisition parameters of the diffusion data underlying this database relative to those used in patients. Furthermore, the deviations were slightly larger for UMCU 1 than for UMCU 2 (paired t_(94)_ = 2.048; p = 0.043). While the predictions for the two databases were highly similar, those of UMCU 2 would be expected to have slightly higher standard deviations due to the decreased number of diffusion volumes, resulting in lower deviations of standard scores for the actual diffusion data of patients.

**Fig. 6:**
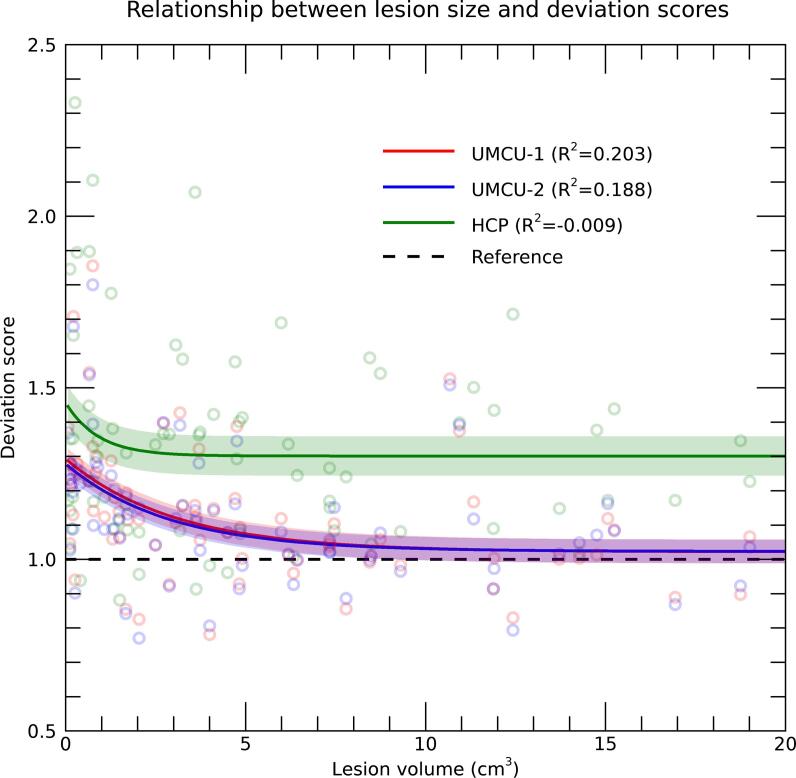
Deviation-scores plotted against the volume of the lesion, with the different colors indicating the different databases used. Each dot is the result of a single patient and database. Solid lines represent the results of an exponential fit, with adjacent shaded areas marking the 95% confidence intervals. The R^2^ in the legend represents the proportion of variance explained by the polynomial fit, adjusted for the number of parameters in the model. The x-axis is truncated at 20 cm^3^ for clearer visualization, excluding 20 patients from the plot (but not the analysis). The horizontal dashed line at a deviation-score of 1.0 is the reference for a fully realistic estimate of standard deviations provided by the prediction.

Importantly, the deviation-scores of the UMCU-2 database indicated that the standard deviations of the predictions underestimate the actual deviations of patient data by approximately 10%. To assess if this underestimation was related to lesion-size, similar as reliabilities, an exponential was fitted to describe the association between lesion-volume and deviation-scores (Fig. 6). Results showed that deviation scores where larger for patients with smaller lesions (approximately ≤ 5 cm^3^), indicating that standard-deviations where primarily underestimated for patients with a smaller lesion extent, while being close to accurate for patients with larger lesions. These results show that the diffusion data of patients fluctuates within the range of the tool's predictions, except for the patients with smaller lesions.

### 3.3 Correlates of stroke induced visual field defects

**Fig. 7:**
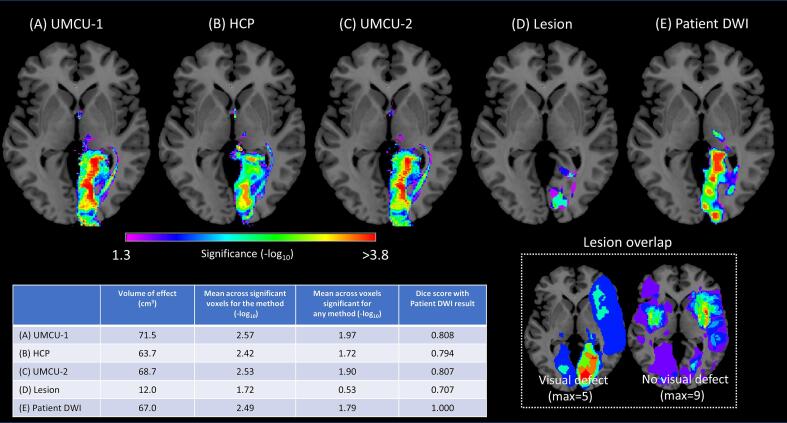
Statistical maps of the comparison between stroke patients with and without visual field defects, with using as input (A) the white matter damage predictions base on the UMCU 1 database, (B) the white matter damage predictions based on the HCP database (C) the white matter damage predictions based on the UMCU 2 database, (D) the lesion volumes, and (E) the fiber count maps according to damaged streamlines as based on the DWI data of the patients. Significant voxels (α = 5%; FWE corrected) are projected on the ch2 single subject T1-weighted image (Rorden and Brett, 2000). Images are displayed according to the neurological convention. The bottom-right part of the image shows the lesion overlap for the group of patients with and without visual field defect. The bottom left of the figure includes the table showing for each method the total volume of voxels with a significant effect, the mean -log_10_ across voxels in this volume for the likelihood the effect was obtained through chance (FWE corrected), and same metric calculated across voxels that were significant for any of the methods.

Results of the comparison between stroke patients with and without visual field defect using the various inputs can be seen in Fig. 7. All analyses based on diffusion data show significant effects in the optic radiations, its projection zone, and the Thalamus. The location of significant voxels for the comparison based on the lesion volumes are restricted to the termination zone of the optic radiations. As a rudimentary analysis of the variations in statistical power between the methods we calculated for each different input the total volume of voxels with a significant effect, the average -log_10_ across voxels with a significant effect for a particular method, and the average -p log_10_ with a significant effect in any of the methods (Fig. 7). The methods based on diffusion data of healthy controls and patients performed roughly similar regarding the volume of significant voxels and their statistical significance. In addition, the dice scores indicated that the overlap of the results based on the patients DWI and those based on the predictions using healthy controls was substantial (Fig. 7). The analysis based on lesion data had a substantially lower volume of significant voxels, and larger likelihoods that the observed effects were obtained through chance.

## 4 Discussion

Indirect structural disconnection mapping is a promising method to predict structural network effects of focal lesions. It requires only a lesion segmentation map to predict tract damage, which makes it efficient in terms of both time and costs. Normative tract-based methods have been applied to map structural networks associated with a variety of functions, with plausible results. The reliability and validity of these indirect methods, however, needs further exploration. In the current study, we present our implementation to predict fiber loss after focal lesions based on healthy control tractography data, that includes estimates of the uncertainty of predictions as an indication of their internal reliability. Moreover, we provide validation of the method by direct comparison with DTI measures derived from patient’s diffusion data. Results show that the tool can predict fiber loss with adequate reliability. The tool's predictions were highly comparable to estimates of fiber loss based on patient’s diffusion MRI, except for cases with small lesions. Healthy control diffusion datasets of different quality were directly compared to investigate the effect of diffusion data quality on the reliability and validity of predictions. The use of high-quality diffusion data improved the internal reliability of predictions as estimated by the procedure. For a proof of concept, we presented a lesion-symptom mapping analysis of visual field defects with lesion location, the tool's predictions or patient’s tractography as input. Results showed a large overlap in results for predictions based on normative DTI and patient DTI data. Tract-based measures, both indirect and direct, outperformed lesion location in statistical power and showed an extended network including optic radiations, its projection zone and thalamus, matching the known anatomy of the visual system (Zhang et al., 2006).These findings demonstrate clinical utility of the method and exemplify the limitations of standard voxel-based lesion-symptom mapping in identifying tract damage after focal lesions.

The use of higher quality diffusion data (HCP dataset) positively affected only the internal reliability, not the external reliability of predictions. The most straightforward explanation for this discrepancy is that the underlying assumption that individual DWI results can be regarded as a gold standard, is incorrect. Individual DWI results will always be approximations of true underlying values due to noise in the acquisition. The more data that can be acquired in single subjects, the better the approximation. This is also reflected in the enhanced similarity of the individual results with the prediction based on the mean of a large group, i.e., the internal reliability, when using the high-quality dataset. As the amount of diffusion data that can be practically acquired in single patients is limited, the noise in individual acquisitions is higher than in the control dataset. We found that the external reliability is no longer affected by increasing the amount of diffusion volumes, indicating that the noise in individual acquisitions is under these circumstances no longer an important factor. The tool's prediction thus to some extent negates the noise in the diffusion data, allowing for more accurate predictions than indicated by the internal and external reliabilities that we estimated. It might therefore be hypothesized that the best approximation of the accuracy of the output of the tool is given by the internal reliability of the HCP database, that would include the least noise in the individual results due to the high field strength at which it was acquired, and the large number of diffusion volumes.

The internal reliability of the prediction is dependent on several factors. These include the size of the lesion, how close the lesion is to the edges of a fiber bundle, and how well the normalization procedure performs in the areas that the damaged fibers are traversing. We reason that the error in the prediction is mainly driven by the inherent limitations of the normalization procedure, and variation in the white-matter structure. Along these lines the effect of lesion size is not surprising, as error in inter-subject alignment following normalization to MNI would impact the overlap of the predictions more for small than for larger lesions. Moreover, we found that for these smaller lesions the internal reliability overestimated the reliability that was attained in patients. A similar effect was observed for standard deviations of the predictions per voxel, which were underestimated for patients with smaller lesions. Theoretically, secondary degeneration or false-negative errors in the manual lesion segmentation could cause a mismatch between results based on patient data and the predictions. However, given the relationship between lesion size and internal reliability, a more speculative explanation is that normalization error is in general larger in patients, either through the lesion itself or other structural abnormalities, thereby disproportionally impacting overlap of smaller lesions, leading to lower reliabilities and higher standard deviations. Nonetheless, estimates of reliabilities and standard deviations of the predictions can be adjusted dependent on lesion size according to this knowledge, for example by weighting scores according to lesion size in prediction models.

Furthermore, in this study, all normalization parameters were established based on anatomical contrast. Alternatively, the between-subject alignment for subjects in the database could have been based on diffusion metrics, e.g., the voxel’s tensor or CSD models (Tournier et al., 2019). While this probably would have improved the inter-subject alignment of tractographies in the database, and thus the estimations of reliability of the tool’s result, this reliability would not be representative when using lesion volumes that are normalized based on anatomical contrast. One potential shortcoming compared to direct measures is the assumption that all axons passing through a lesion area are non-functional as a result of the stroke. This assumption may not be completely correct, as abnormal contrast in FLAIR images may not always signify a full functional disconnection. The patient’s DTI data also showed that changes to the structural integrity of the white matter did not always prevent tracking through voxels that were marked as part of the lesion (meaning that FOD values surpassed the tracking threshold while angles of streamlines were not abnormal). For the comparison with the tool's predictions, all streamlines in patients within the lesion area were assumed damaged. The tool results may thus include false positives. Note, however, that this issue is not specific to our tool, it is inherent to all approaches to lesion-symptom mapping. A second shortcoming is that assigning a single value of disconnection to a voxel ignores the putative presence of crossing fibers, where some axons transversing a voxel may be disrupted, while others are not. The voxel-level visitation maps represent streamline density for each voxel. Voxels that have the same non-zero fiber-count across multiple subjects could still vary in the fiber orientations that they enclose. This can also lead to false positive predictions of disconnection, attenuating the strength of the link with clinical lesion symptoms. There are other approaches to disconnection mapping that estimate streamline loss at the level of macroscale white matter tracts or use connectivity matrices to represent streamline loss between grey-matter parcels (Gleichgerrcht et al., 2017, Griffis et al., 2021, Kuceyeski et al., 2015). The benefit of voxel-level visitation maps is that they are easy to use as input for second-level group analyses. This makes it a popular approach, that can used in combination with tract-level measures. Last, we used two sets of healthy controls diffusion weighted imaging datasets to build three normative tractography databases. These included a large set (n = 173) of high-resolution diffusion imaging data derived from the HCP data and a second set of 78 healthy control subjects acquired at the University Medical Center Utrecht. The HCP subjects were mostly young adults and thus differ substantially from our middle to old age stroke cohort. The age range of the UMCU cohort was wider, though the median age remained younger than of the stroke cohort. For an optimal comparison, demographics of the control datasets should have been matched to that of the patients. Previous studies did, however, indicate that normative disconnection maps have a very high reproducibility across age decades in adulthood (age 21 and above) (Foulon et al., 2018, Thiebaut de Schotten et al., 2020) and the same HCP dataset has been used in prior disconnection mapping studies (Alves et al., 2021, Billot et al., 2022). Also, the impact of age-related differences would be in the direction of an underestimation rather than overestimation of reliability.

Several research groups have advocated the importance of including estimates of white matter damage in lesion-symptom mapping (Corbetta et al., 2015, Grefkes and Fink, 2014, Reber et al., 2021). The theoretical underpinnings are clear. Lesions at different locations can damage an axon at different points along its structure, while still resulting in the same clinical symptoms. Moreover, disconnection at one location doesn’t have a lower impact than demolishment of the entire tract. Hence, using predictions of tract damage to establish lesion-symptom associations has less variability in individual voxels and a higher power to detect true associations. There are several software tools now available that use normative connectivity data to estimate structural disconnection (Foulon et al., 2018, Griffis et al., 2021, Kuceyeski et al., 2015). Recent papers illustrated the potential of this approach (Biesbroek et al., 2021, Griffis et al., 2019, Kuceyeski et al., 2016, Thiebaut de Schotten et al., 2020) and a first validation study for structural disconnection mapping was recently published (Wawrzyniak et al., 2022). In their validation, the authors reproduced the known association between corticospinal tract damage and hemiparesis, similar to what our findings demonstrated for hemianopsia and the optic radiations and their projections. Nonetheless, studies contrasting models based on lesion location versus structural connectivity measures to predict clinical outcome, show discrepant results. Where some studies report superiority of (adding) connectivity measures (Bowren et al., 2022, Del Gaizo et al., 2017, Wawrzyniak et al., 2022), others find no difference between lesion and structural disconnection maps (Hope et al., 2018, Salvalaggio et al., 2020). Thus, while the theoretical advantages of structural disconnection mapping are well-defined, the additive value of disconnection measures in outcome prediction models is still questioned. Methodological variability between existing tools is likely to have a role in these inconsistencies, since the tools vary in tracking method, the normative data that is used, and the output measures that are calculated. Lesion methods, including indirect measures, have many degrees of freedom (Sperber and Karnath, 2018) and variability in the analysis is a known problem in the neuroimaging field (Botvinik-Nezer et al., 2020). It is also important to consider that these differences may not be directly attributed to the indirect disconnection approach itself. The impact of structural disconnection may vary depending on the function of interest, the sample composition and within-sample lesion distribution. Findings from this paper demonstrate the impact of lesion size of the sample under study. The sample from Corbetta et al. (2015) that is used in the paper by Salvalaggio and collegues (2020) has a relatively small average lesion volume compared to the current stroke dataset (3.4 ml versus 17.1 ml). Small and distinct lesions are favorable in terms of spatial specificity but have a higher prediction error in indirect methods. Hence, this may provide one explanation why structural disconnection maps did not perform better than lesions in predicting behavior in their sample (Salvalaggio et al., 2020).

In conclusion, indirect measures of structural disconnection are a reliable and valid substitute for tractography based on patient’s DWI for estimations of fiber loss. Analyses using predictions based on a healthy control database would accomplish similar results as when using diffusion data of patients, which saves substantial costs and effort. Moreover, when using high quality datasets, the tool may even be preferable above (lower quality) single subject diffusion MRI. Further work is needed to elucidate whether models using (indirect) connectivity measures also outperform models using only traditional lesion characteristics in predicting behavior, cognition, and clinical symptoms.

## Funding sources

The FAB4V study and A.S. and E.H. were supported by an European Research Council (ERC) Advanced Grant (#339374).

## Sharing data

The code used in this study is available through a GitHub repository (https://github.com/UMCU-RIBS/simDTI). The results can be downloaded from Mendeley Data (Smits, Anouk; Raemaekers, Mathijs (2023), “Reliability and validity of DTI-based indirect disconnection measures”, Mendeley Data, V1, https://doi.org/10.17632/ggzr68kmrt.1).

## CRediT authorship contribution statement

**A.R. Smits:** Conceptualization, Methodology, Investigation, Formal analysis, Writing – original draft. **M.J.E. van Zandvoort:** Resources, Writing – review & editing, Supervision. **N.F. Ramsey:** Conceptualization, Writing – review & editing, Supervision. **E.H.F. de Haan:** Resources, Writing – review & editing, Supervision, Funding acquisition. **M. Raemaekers:** Conceptualization, Methodology, Formal analysis, Software, Writing – original draft.

## Declaration of Competing Interest

The authors declare that they have no known competing financial interests or personal relationships that could have appeared to influence the work reported in this paper.
