# Supplementary material for: Reliability and validity of DTI-based indirect disconnection measures
Source: Neuroimage Clin. 2023 Jul 11;39:103470. doi: 10.1016/j.nicl.2023.103470 (PMC10368919; doi:10.1016/j.nicl.2023.103470)

# Supplementary material

*Supplementary Figure 1.*

Lesion prevalence map superimposed on the MNI brain. Multiple slices are shown in the axial, sagittal and coronal plane. The legend refers to the number of patients with a lesion at that voxel, with dark red indicating a higher number of patients. The maximum number of patients with an overlap of lesion is 12/95. L = left, R= right.


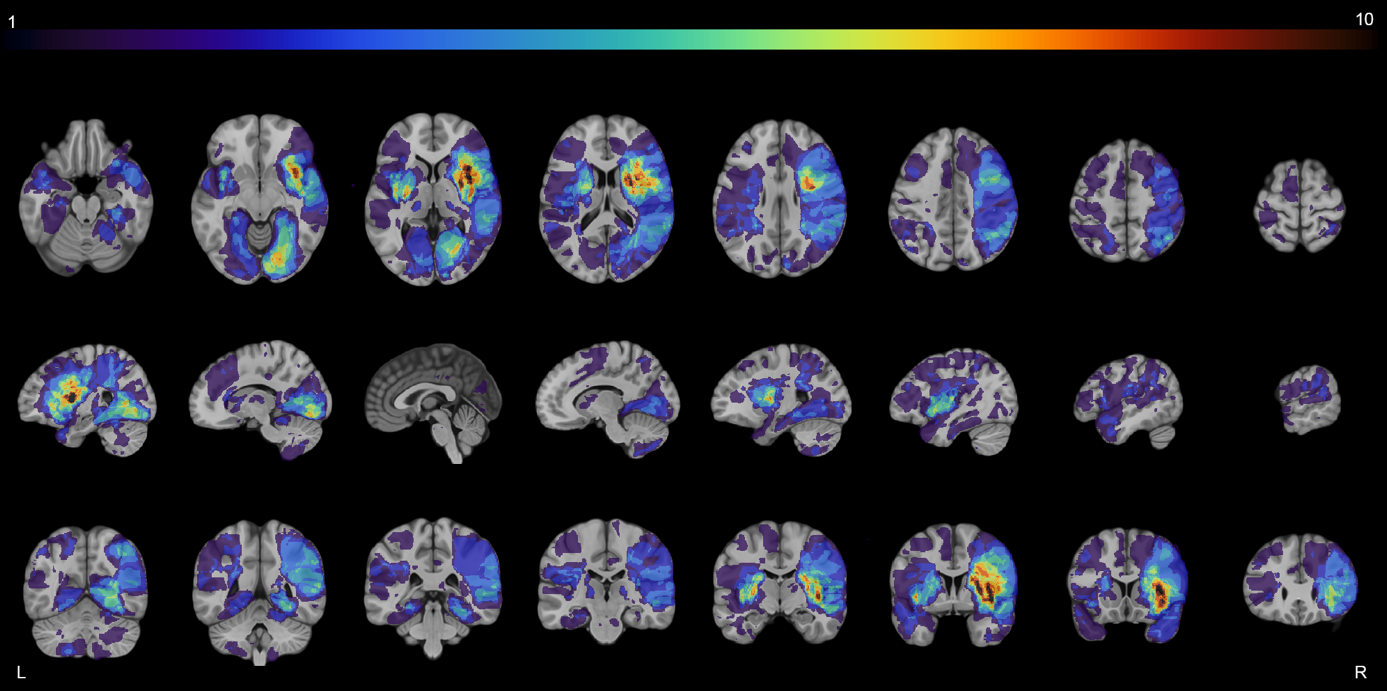

Supplement: Supplementary data 1 [file mmc1.docx]
